# Exploring glioblastoma stem cell heterogeneity: Immune microenvironment modulation and therapeutic opportunities

**DOI:** 10.3389/fonc.2022.995498

**Published:** 2022-09-21

**Authors:** Amanda L. Johnson, John Laterra, Hernando Lopez-Bertoni

**Affiliations:** ^1^ Hugo W. Moser Research Institute at Kennedy Krieger, Baltimore, MD, United States; ^2^ Department of Neurology, Johns Hopkins University School of Medicine, Baltimore, MD, United States; ^3^ Department of Neuroscience, Johns Hopkins University School of Medicine, Baltimore, MD, United States; ^4^ Department of Oncology, Johns Hopkins University School of Medicine, Baltimore, MD, United States

**Keywords:** cancer stem cell, immunomodulation, multi omics, cancer therapy, cellular mimicry, spatial analysis, single-cell sequencing

## Abstract

Despite its growing use in cancer treatment, immunotherapy has been virtually ineffective in clinical trials for gliomas. The inherently cold tumor immune microenvironment (TIME) in gliomas, characterized by a high ratio of pro-tumor to anti-tumor immune cell infiltrates, acts as a seemingly insurmountable barrier to immunotherapy. Glioma stem cells (GSCs) within these tumors are key contributors to this cold TIME, often functioning indirectly through activation and recruitment of pro-tumor immune cell types. Furthermore, drivers of GSC plasticity and heterogeneity (e.g., reprogramming transcription factors, epigenetic modifications) are associated with induction of immunosuppressive cell states. Recent studies have identified GSC-intrinsic mechanisms, including functional mimicry of immune suppressive cell types, as key determinants of anti-tumor immune escape. In this review, we cover recent advancements in our understanding of GSC-intrinsic mechanisms that modulate GSC-TIME interactions and discuss cutting-edge techniques and bioinformatics platforms available to study immune modulation at high cellular resolution with exploration of both malignant (i.e., GSC) and non-malignant (i.e., immune) cell fractions. Finally, we provide insight into the therapeutic opportunities for targeting immunomodulatory GSC-intrinsic mechanisms to potentiate immunotherapy response in gliomas.

## 1 Glioblastoma stem cells

**Figure 1:**
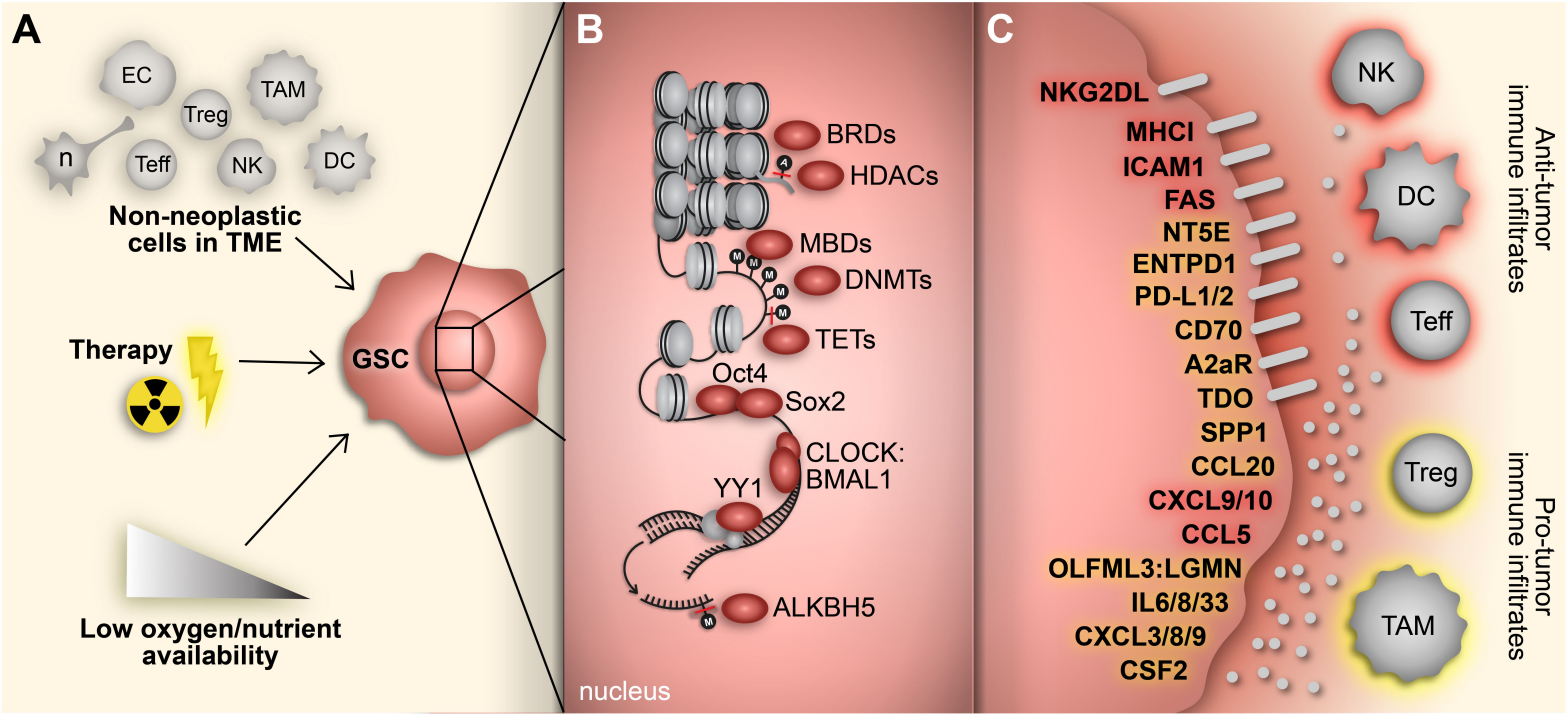
Adaptive responses drive immunosuppressive GSC mechanisms. **(A)** GBM cells face constant adaptive pressure due to stimuli in the surrounding TME including cell-cell interactions with non-neoplastic counterparts, standard-of-care therapeutics and availability of oxygen and nutrients. **(B)** These extrinsic stimuli induce reprogramming events, including acquisition of a stem-like state in GBM cells. Transition to a GSC state results from altered gene expression mediated by various transcriptional regulators (e.g., epigenetic modifiers, transcription factors) that regulate immunomodulatory mechanisms. **(C)** GSC-intrinsic immunomodulatory mechanisms occur through induction (yellow) or repression (red) of immune-related genes that play a key role in shaping the TIME in GBM. Cell types: EC, endothelial cell; n, neuron; Teff, effector T cell; Treg, regulatory T cell; NK; natural killer cell; TAM, tumor-associated macrophage/microglia; DC, dendritic cell.

High-grade gliomas, including glioblastoma (GBM), are highly heterogeneous with a complex oncogenic microenvironment consisting of distinct tumor niches and remarkable cell heterogeneity (1, 2). A critical component of glioma malignancy derives from the distinct population of glioma stem cells (GSCs) that function to promote and maintain oncogenicity through their capacity for self-renewal, cellular adaptation, and multipotency (3–5). These stem-like cells engage in a synergistic relationship with the surrounding tumor microenvironment (TME) to promote tumor progression and contribute to the vast degree of intratumoral heterogeneity, immune-suppression, and therapy resistance encountered in gliomas (5). The plasticity of GSCs facilitates shifts between distinct tumorigenic stem-like states and allows for transitions along the spectrum of differentiation that characterizes glioma cells (6–8). These state transitions are brought on by various stemness-inducing stimuli (e.g., hypoxia, interaction with non-neoplastic cells, immune exposure, therapeutics, etc.) and carried out by reprogramming mechanisms that involve specific transcription factors and dynamic epigenetic modifications that alter transcriptional profiles and consequently, cellular and molecular phenotypes (9) (**Figure 1**).

A unique facet of cancer stem cell biology is the capacity of a tumor-propagating stem-like cancer cells to evolve from a non-stem-like state through a process known as dedifferentiation. Reprogramming transcription factors, collectively referred to as the Yamanaka factors (Oct3/4, Sox2, Klf4, c-Myc), play critical driving roles in this process (10, 11). In particular, Oct4 and Sox2 are sufficient to induce stem cell properties and *in vivo* tumor-propagating potential in differentiated and non-tumor-propagating GBM cells (12, 13). Additionally, our lab has identified multiple mechanisms downstream of Oct4 and Sox2 underlying GSC stemness and tumorigenicity (12–17). Furthermore, epigenetic modifications mediated by Sox2 and Oct4 have implications in GSC-immune interactions that contribute to an immunosuppressive TME (17).

In spite of the progress in our understanding of GSC biology, the inherent heterogeneity and plasticity of the GSC population and resulting phenotypic consequences have restricted the impact of ongoing therapeutic efforts. In this review, we cover the advancements in our understanding of GSC plasticity, GSC-intrinsic immunomodulatory mechanisms, and the capacity of GSCs to co-opt immunosuppressive cell phenotypes. Additionally, we discuss the application of evaluable and emerging cutting-edge techniques to study immunosuppressive GSC states and cell-cell interactions at high cellular resolution. Finally, we discuss potential therapeutic approaches aimed at exploiting epigenetic mechanisms and metabolic vulnerabilities associated with acquired immunosuppressive GSC states to enhance the efficacy of GBM immunotherapy.

## 2 Contribution of GSCs to the immunosuppressive microenvironment in glioblastoma

### 2.1 Immunosuppressive TME in GBM

A hallmark of GBM and significant barrier to immunotherapies is the immunosuppressive TME defined by relatively high numbers of suppressive, pro-tumor immune cell infiltrates (e.g., regulatory T cells (Tregs), tumor-associated microglia, tumor-associated macrophages (TAMs), myeloid-derived suppressor cells (MDSCs)) and high prevalence of dysfunctional T cell states (e.g., exhaustion) (18–26). High infiltration of immunosuppressive myeloid and lymphoid cell populations negatively correlates with patient prognosis and therapy response in GBM (19, 25, 27–29). Notably, most infiltrating immune cells in GBM are of myeloid origin (e.g., monocyte-derived macrophages, brain-resident microglia, and MDSCs) and are defined by a variety of pro-tumor phenotypes that function to suppress the anti-tumor immune response (20, 24, 26, 30–32). Beyond their direct immune suppressive functions, pro-tumor myeloid populations (i.e., TAMs, microglia, and MDSCs) promote GBM growth, invasion, and angiogenesis (33–39). Moreover, TAMs have the capacity to induce a mesenchymal state in GBM, which is associated with therapy resistance and poor patient survival (40–42).

As mentioned above, GBM is highly heterogeneous and can be broadly classified by three molecularly defined subtypes – classical, proneural, or mesenchymal (43) – each with unique immunosuppressive characteristics. The diversity of myeloid cell states is demonstrated by the existence of myeloid phenotypes that preferentially associate with specific GBM subtypes and specific tumor niches. For example, mesenchymal tumors are enriched in blood-derived macrophages that possess a transcriptionally distinct immunosuppressive profile marked by upregulation of genes involved in chemokine signaling and lymphocyte chemotaxis and reside in microvascular and peri-necrotic regions (31).

GSCs also avoid recognition by the immune system through expression of checkpoint inhibitors, including PD-L1, CD70, A2aR and TDO, and downregulation of antigen-presentation molecules, specifically MHC class I (MHC-1) molecules (17, 44–46). Furthermore, GSCs can regulate the immune TME by recruiting and polarizing myeloid cells to an immunosuppressive state, producing T cell-suppressing cytokines, and monopolizing nutrients necessary for proper T cell function (47–51). For example, Wu et al. demonstrated GSC-mediated recruitment and polarization of macrophages and microglia to an M2-like phenotype through production of TGF-β1, MIC-1 and CSF-1 (47). From a metabolic standpoint, GSCs preferentially take up glucose from the microenvironment, leading to impaired T cell function due to glucose deprivation (49–51).

There are multiple contributors to the lack of immunotherapy efficacy in GBM. These include a relatively low gene mutation rate resulting in relatively few tumor-associated neoantigens (52), a unique TME comprised of an abundance of tumor-associated immunosuppressive M2-like macrophages (comprising up to 60% of immune cells) (20) and the immune-privileged brain environment (53). However, the fact that standard-of-care chemo/radiation for newly diagnosed GBM can lead to hypermutated recurrent tumors (54) unresponsive to immunotherapy and the efficacy of immunotherapy in cases of CNS metastases (e.g., melanoma) emphasize the potential dominant role for the GBM TME in driving immunotherapy resistance (55–58). For example, the TME of brain metastases and primary CNS tumors, including GBM, have distinct immune landscapes whereby brain metastases contain higher infiltration of leukocytes and fewer cells of monocytic-lineage (19).

### 2.2 Molecular regulation of the immunosuppressive GSC transcriptome

Understanding the therapeutic consequences and vulnerabilities of GBM cell heterogeneity and plasticity is an ongoing effort in the scientific community. Despite the association between genetic aberrations and cell states in GBM (59), genomic alterations alone are insufficient to explain the highly adaptive and heterogeneous nature of GSCs (60). Epigenetic modifications mediate the acquisition of a stem-like state in cancer cells, including GBM, and recent studies have implicated epigenetic variability as a driving force in the adaptation of GBM to external stimuli throughout tumor evolution (4, 12, 14–16, 60–62).

Epigenetic mechanisms, including the addition and removal of activating or repressive marks to histone proteins, DNA, and RNA, serve to regulate gene expression in a reversible manner (62, 63). Notably, the balance between transcriptionally repressive methylation and activating demethylation of DNA, controlled by DNA methyltransferases (DNMTs) and ten-eleven translocation proteins (TETs), respectively, governs acquisition and maintenance of the GSC phenotype (12, 16). On the other hand, RNA methylation occurs post-transcriptionally to alter mRNA stability and impact stem cell differentiation (64–66). Histone proteins, which are essential for forming and stabilizing chromatin structure in either accessible (euchromatin) or inaccessible (heterochromatin) states, are also susceptible to modifications (e.g., methylation, acetylation, phosphorylation) which consequently alter chromatin architecture and lead to activation or repression of gene transcription (63). The progression from chromatin remodeling to transcription activation involves the activity of epigenetic readers, such as bromodomain and extraterminal domain (BET) proteins, that detect and bind histone marks. Readers recruit transcription regulatory factors and/or chromatin remodeling protein complexes to control gene expression (67). Collectively, dysregulation of epigenetic factors underlies the acquisition, maintenance, and plasticity of the GSC phenotype and represents a promising avenue for GSC-targeted therapeutics in GBM.

A relatively underappreciated aspect of dynamic GSC states is their contribution to the immunosuppressive tumor microenvironment. The role of the tumor microenvironment, specifically the immune compartment, in driving GBM and GSC states is being increasingly elucidated (7, 31, 41, 68, 69). However, improved understanding of how GSC adaptations influence the immune TME is crucial to overcome the immunosuppressive pressures that diminish the efficacy of immunotherapy in gliomas. As discussed above, the cell-intrinsic factors that modify and drive GSC states and transitions are abundant and include specialized reprogramming transcription factors, epigenetic modifiers, transcriptional regulators, cellular cycles, etc. In this section, we discuss findings that highlight the effects of GSC-intrinsic transcriptional variation and cellular transitions on their immunomodulatory role.

#### 2.2.1 Transcription factors

As mentioned previously, the most notable transcription factors associated with acquisition of a stem-like phenotype are the Yamanaka factors. Of particular interest in the context of glioma, especially related to the immune influence of GSCs, are Sox2 and Oct4. Sox2, which is a ubiquitously expressed transcription factor in cancer stem cells, has a direct effect on the immunosuppressive capacity of GSCs through induction of CD39 (ENTPD1), an ectonucleotidase responsible for hydrolyzing ATP towards adenosine and a critical mediator of immune response in cancer (70, 71). In GSCs, knockdown of Sox2 increased the extracellular ATP concentration and enhanced dendritic cell recruitment and phagocytic-ability as well as T-cell-mediated GSC lysis in co-cultures in direct association with a reduction in CD39 (70).

Another noteworthy stem cell-driving transcription factor, Oct4 (POU5F1), cooperates with Sox2 to induce an immunosuppressive transcriptome in GSCs, defined by induction of immune checkpoint inhibitory molecules (PD-L1, CD70, A2aR and TDO) alongside dysregulation of immune modulatory cytokines and chemokines including upregulation of SSP1, IL8, CXCL3, and CCL20 and downregulation of CCL5, CXCL9, and CXCL10. This GSC immune-suppressive phenotype was found to be mediated by and dependent on the BET protein BRD4 which is involved in directing chromatin remodeling in response to histone modifications (17).

Aside from the classical cancer stem cell reprogramming transcription factors, other transcription factors are involved in both the induction of a glioma stem-like state and acquisition of an immunosuppressive GSC phenotype. Yin Yang 1 (YY1), a zinc-finger transcription factor involved in polycomb protein recruitment and transcription regulation (72, 73), is necessary for maintenance of the stem cell phenotype in GBM, is associated with Sox2 and Oct4 expression across cancers (74–77), and mediates both chemotherapy and radiation resistance in GBM (74, 78). The YY1-CDK9 transcriptional complex in GBM cells promotes Treg infiltration, inhibits RNA methylation-dependent interferon responses, and reduces the efficacy of immune checkpoint inhibitor therapy in GBM (77), highlighting how transcription factors and epigenetic modifications cooperate to impart immunomodulatory function in GBM cells.

While we typically think of external pressures when discussing induction of stemness, pre-programmed endogenous processes such as the circadian rhythm may also drive and maintain the stem cell phenotype in cancer cells (79, 80). The circadian rhythm is a cell-autonomous, cyclical process composed of transcriptional-translational feedback loops that govern carefully timed adjustments in gene expression (81). The transcriptional variation attributed to circadian oscillations has a demonstrated capacity to alter cancer stem cell states, their interaction with the TME, and their susceptibility to therapeutic agents (79, 82–86). Disruption of the circadian rhythm leads to altered immune cell infiltration and T-cell exhaustion in a variety of cancers (85, 87, 88). In glioma, the CLOCK-BMAL1 complex, the main transcriptional component in the circadian rhythm, modulates the stem cell phenotype by enhancing self-renewal capacity and migration and regulating GSC metabolism (89–91). Furthermore, CLOCK, in partnership with BMAL1, upregulates LGMN *via* induction of OLFML3, a novel microglia-recruiting factor, in GSCs to enhance infiltration of microglia and polarize them to an immunosuppressive phenotype (89, 92). This finding, along with our understanding of circadian dysfunction in cancer, demonstrates the possibility of dynamic immunomodulatory GSC phenotypes mediated by cell-intrinsic processes.

#### 2.2.2 Histone modifications

As discussed previously, readers of histone modifications, e.g., BRDs, can direct acquisition of an immunosuppressive phenotype in GSCs (17). BRDs also have an immunomodulatory role in ATRX-deficient, IDH-mutant gliomas. Deficiency of ATRX, a histone protein involved in the chromatin remodeling SWI/SNF family, occurs in >80% of IDH-mutant grade II/III astrocytomas and >50% of secondary GBMs (93, 94). Hu et al. reports a BRD3/4-dependent induction of an immunosuppressive transcriptome, marked by upregulation of PD-L1/2, IL-33, CXCL8/9, CSF2, and IL-6, in ATRX-deficient glioma cells (95). Furthermore, this ATRX loss resulted in decreased T-cell-mediated glioma cell lysis, increased macrophage immune-suppressive M2-like polarization, and enhanced Treg tumor infiltration. Independently, Babikir et al. found increased infiltration of immunosuppressive monocyte-lineage cells in ATRX-mutant versus ATRX-wildtype IDH-mutant glioma (96). While the former findings were not in the context of cancer stem cells, ATRX-mutant IDH1-mutant GSCs have been shown to possess increased cell propagation capacity and upregulation of TGF-beta-associated pathways compared to ATRX-wildtype GSCs (97) suggesting that ATRX-deficiency enhances certain stemness characteristics in glioma in accordance with increased immunosuppressive effects.

Enzymes that remove histone acetylation marks, known collectively as histone deacetylases (HDACs), also regulate GSC-intrinsic immunomodulatory mechanisms (44, 98). A major factor in the poor immunogenicity of gliomas derives from the innate ability of GSCs to evade the immune response through dysregulation of major histocompatibility complex (MHC) molecule expression (44). Specifically, Yang et al. demonstrated HDAC-dependent MHC-1 downregulation in GSCs that consequently suppressed the function of tumor-infiltrating lymphocytes (TILs). Furthermore, pan-HDAC inhibition not only enhanced MHC-1 expression and the T-cell response, but it downregulated Sox2 and Oct4 protein expression and decreased the self-renewal capacity of GSCs. While the knowledge that HDAC inhibitors upregulate MHC-1 molecule expression in cancer is not new (99–103), this study extended the findings to gliomas and, more importantly, detailed the association between HDAC regulation of MHC1 expression and maintenance of the stem cell phenotype. Aside from MHC-1 expression, histone deacetylation *via* HDAC8 in glioma cells represses NKG2D ligand expression to evade natural killer (NK) cell-mediated tumor cell death (104). A study by Zhan et al. found that HDAC1 and HDAC2 promote GSC evasion of the suppressive interferon-mediated immune response through their involvement in the nucleosome remodeling and deacetylase (NuRD) complex (105). Zhan and colleagues show that methyl-CpG-binding domain 3 (MBD3) recruits and assembles the HDAC-containing NuRD complex at the promoter of STAT1 where deacetylation of H3K27 leads to repression of STAT1 transcription. From there, STAT1 repression desensitizes GSCs to interferon treatment. The driver of this mechanism, MBD3, also regulates proliferation, viability, and self-renewal capacity of GSCs and positively correlates with expression of Sox2, Olig2, and Nestin in GBM specimens (106). Together, these findings directly link glioma cell-intrinsic HDAC-mediated immunosuppression to the acquisition and maintenance of the stemness phenotype of GSCs.

#### 2.2.3 DNA and RNA methylation

The dynamics and consequences of DNA methylation in cancer biology have been vastly explored. Patterns of DNA methylation mediate repression of gene transcription thereby altering cell states and phenotypes and contributing to tumor progression (12, 16, 60). In the context of gliomas, levels of DNA methylation are associated with the IDH-mutation status of the tumor with IDH-mutant gliomas tending to acquire a hypermethylated phenotype, commonly referred to as the glioma CpG island methylator phenotype or G-CIMP (107). This CpG island hypermethylation has been implicated in controlling immunomodulatory effects of GSCs in both IDH-mutant and IDH-wildtype gliomas. Specifically, in IDH-mutant GSCs, increased methylation of the NKG2D ligand gene promoter suppresses its expression leading to the ability of GSCs to evade NK cell-mediated cytotoxicity (108). In line with evidence that decreasing DNA methylation in cancers enhances immunogenicity through upregulation of MHC-1 expression (109–112), GSCs are found to contain increased methylation along the regulatory regions of HLA genes (113). Similarly, a separate study discovered that methylation-dependent regulation of MHC-1 and ICAM1 expression regulates T cell-mediated GSC cytotoxicity in IDH-wildtype glioma (46). Gangoso et al. demonstrated emergence of a DNA methylation-dependent immune evasive phenotype highly associated with mesenchymal and mesenchymal-like GBM states (68). These GSCs were found to have extensive hypomethylation, especially along genes involved in immune-suppressive processes. Furthermore, immune evasive GSCs fostered an immunosuppressive TME *in vivo* characterized by enhanced infiltration of macrophages and myeloid-derived suppressor cells (MDSCs) and increased TIL dysfunction (68).

Unlike DNA methylation, RNA methylation is a relatively underappreciated regulator of the stem cell phenotype. In embryonic stem cells (ESCs), low levels of N (6)-methyladenosine (m6A methylation) promote pluripotency and protect against differentiation (64–66). Many transcripts of pluripotency genes, including Nanog, Sox2 and Klf2, are regulated by m6A methylation in both human and mouse ESCs (64, 65). In GSCs, Cui et al. found that METTL3, a catalytic subunit of the m6A methyltransferase complex, reduces cell proliferation and self-renewal capacity along with CD44 expression, through methylation of pluripotency-driving transcripts (114). However, conflicting roles of METTL3 in GSCs have been demonstrated by other studies (115–117). Visvanathan et al. showed that GSCs are enriched for METTL3 which functions to stabilized SOX2 expression and enhance self-renewal capacity (117). Moreover, METTL3 has been linked to TMZ resistance in GBM cells (115, 116). On the other hand, ALKBH5, an m6A RNA demethylase, is highly expressed in GSCs and associated with poor patient prognosis in GBM (118). Through demethylation, ALKBH5 enhances both self-renewal capacity and expression of stemness genes (i.e., Nestin, Sox2, Nanog, and Oct4) in GSCs (118). Furthermore, ALKBH5 can indirectly regulate the GBM cell-intrinsic immunosuppressive phenotype by demethylating the lncRNA NEAT1 which allows for NEAT1-mediated paraspeckle assembly (119). From there, relocation of transcriptional repressors to the newly assembled paraspeckles results in upregulation of CXCL8 and IL8. Consequently, ALKBH5-initiated CXCL8/IL8 induction improves tumor-associated macrophage infiltration and promotes tumor progression, demonstrating the impact of post-transcriptional epigenetic modifications in regulating the immunosuppressive GSC phenotype. These findings suggest the need for a more refined investigation of the role of m6A methylation in controlling the GSC phenotype, taking special care to account for and address the heterogeneous and dynamic states of GSCs.

#### 2.3 Immunosuppressive influence of metabolic plasticity in GSCs

A hallmark of cancer, metabolic remodeling permits cells to adapt and persist in conditions of depleted oxygen and/or nutrients by utilizing alternative metabolic pathways (120). Cancer stem cells in particular possess a great potential for metabolic reprogramming compared to more differentiated counterparts (121–123). In GBM, GSCs readily adjust their metabolic preferences to meet their energy demands (49, 123–125). Remodeling in response to hypoxic or low glucose environments occurs through various mechanisms including epigenetic modification (3, 126, 127), HIF-1α signaling (125, 128, 129), and altered expression of metabolic enzymes and transporters (49, 130). In general, metabolic profiles in GBM cells are spatially distinct and attributed to TME interactions and restricted nutrient availability (122, 125, 131, 132) with different GSC subsets having distinct metabolic preferences and dependencies (121, 133, 134).

Notably, metabolic reprogramming in GSCs modulates the anti-tumor immune response in favor of tumor progression. As mentioned previously, GSCs suppress T cell function and recruit and polarize microglia through glucose monopolization and circadian dysregulation, respectively (49, 89, 90). Additionally, GBM cells in spatial proximity to immune cells have distinct metabolic profiles associated with immune-suppressive transcriptional signatures. Similar to immune-responsive cells, hypoxia-responsive GBM cells, which are enriched for GSCs, reside in regions enriched in TAMs and T cells (including exhausted CD8^+^ T cells), linking hypoxia-induced metabolic remodeling to tumor-promoting immune interactions (135). Furthermore, Coy et al. identified increased levels of extracellular immune-suppressive adenosine in GBM attributed to high expression of CD73, which functions alongside CD39 to metabolize ATP into adenosine, in hypoxia-responsive GBM cells (131). Collectively, these findings demonstrate a connection between TME-driven metabolic reprogramming and GSC-mediated immune suppression, and prompt further investigation into therapeutically relevant metabolic vulnerabilities in these immune-suppressive cells.

#### 2.4 GSC plasticity and immune cell mimicry

**Figure 2:**
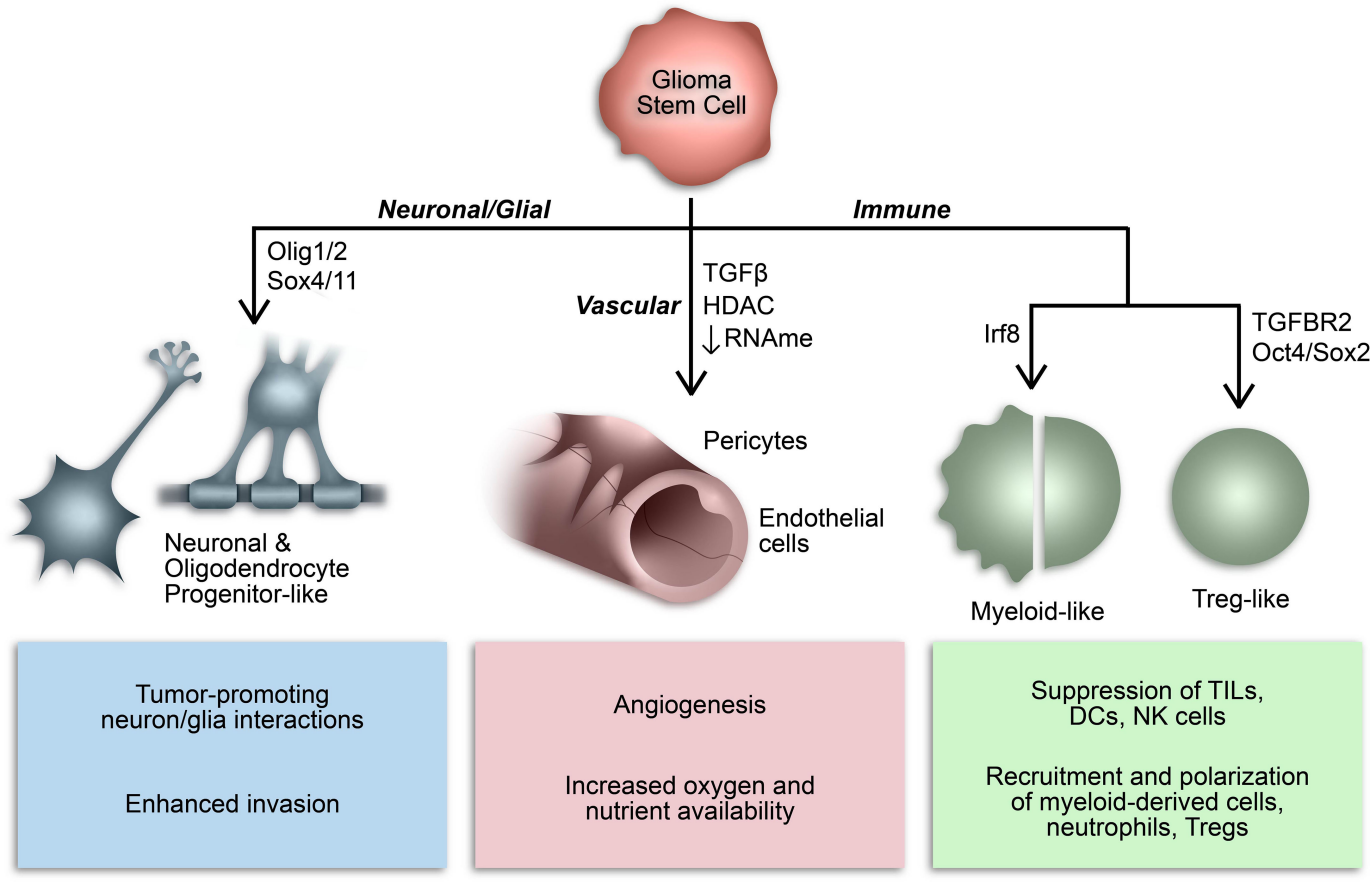
Consequences of GSC lineage plasticity. A defining characteristic of GSCs is their multipotency, or ability to differentiate into various cell types within a particular cell lineage. Cancer stem cells, including GSCs, can imitate cell types from multiple lineages by co-opting their transcriptional profiles and consequently mimicking their functions.

Over the last decade or so, our understanding of the phenotypic plasticity of cancer cells has expanded to include the ability of cancer stem cells to behave like, or mimic, the function of other cells (**Figure 2**) (136). For example, GSCs can behave like vascular endothelial cells and pericytes to promote angiogenesis and increase oxygen and nutrient availability in a phenomenon referred to as vasculogenic mimicry (137–139). In many respects, this epitomizes the inherent plasticity of GSCs and is mediated by epigenetic mechanisms and influenced by conditions in the TME (136–141). Additionally, subsets of GSCs resemble neuronal and glial progenitor cells and engage in synaptic interactions to promote tumor invasion and progression (59, 142–146). Notably, a similar process affecting the GBM immune microenvironment has been identified whereby GSCs mimic immune cell function by co-opting transcriptional profiles typically associated with myeloid cells (68, 147). The aforementioned study by Gangoso et al. demonstrated acquisition of a myeloid-related immunosuppressive transcriptional profile in GSCs mediated by a DNA methylation-dependent immunoediting mechanism in response to repeated immune exposure (68). In particular, *Irf8*, *Nt5e*, and *Cd274* were among the genes upregulated in immune evasive, mesenchymal-like GSCs due to DNA demethylation. Due to the often myeloid-specific expression of *Irf8* and transcriptome-level induction of myeloid-related signatures, they concluded that altered DNA methylation governs acquisition of a myeloid-mimicking phenotype in GSCs (68). Furthermore, our lab has identified a Treg-like transcriptional profile in *TGFBR2* ^high^ GSCs driven by Oct4 and Sox2 that regulates the immunosuppressive TME by repressing T cell function through the action of effector genes canonically expressed by Tregs (e.g., CD274, ENTPD1, NT5E, LGALS1, TGFB1) (148). Collectively, these findings suggest that GSCs co-opt functions of non-neoplastic cells from the TME through dynamic state transitions to facilitate tumor progression and suppress the immune response.

In summary, GSCs alter their immunomodulatory phenotype in response to stemness-regulating stimuli (**Figure 1**). A variety of gene expression regulators carry out these transformations, from pluripotency-driving transcription factors and circadian transcriptional complexes to epigenetic modifications (**Table 1**). While improving, our understanding of the immunosuppressive functions of GSCs as they relate to their heterogeneous and ever-changing states is limited. Unfortunately, many studies conducted in the context of GSC-immune interactions are restricted from the start to certain subpopulations of GSCs (e.g., gatekeeping GSCs by their CD133 expression). This not only sets the stage for potentially context-dependent findings, but inaccurately represents the diverse spectrum of GSC phenotypes found in patient specimens. Moving forward, it is essential to approach studies of GSC-intrinsic mechanisms from an unbiased starting point, especially in the context of TME interactions whereby both cell intrinsic and extrinsic influences constantly drive GSC adaptation and state plasticity. In the next section, we discuss cutting-edge techniques and bioinformatics tools available to study heterogeneous and dynamic GSC states at a high cellular resolution allowing for deep exploration of the bidirectional interaction between GSCs and cells of the TME that influence GSC-mediated immunomodulation (**Table 2**).

**Table 1:** GSC-intrinsic mechanisms modulating the immune response.

Class	Regulatory Factor	Mechanism	Immune effect	GSC context	Ref(s)
Transcription Factors	Sox2	Decreased extracellular ATP *via* CD39 induction	↑ DC-mediated GSC phagocytosis; ↓ T cell-mediated GSC lysis	Patient-derived GSCs	(70)
	Oct4 (*POU5F1*) and Sox2	BRD4-dependent induction of checkpoint inhibitory molecules and immune-suppressive cytokines/chemokines	↓ T cell infiltration, ↑ Treg infiltration, ↓ T cell activation, ↑ M2-like macrophage polarization	Patient-derived GSCs	(17)
	Yin Yang 1 (*YY1*)	Facilitates m6A-mediated suppression of interferon-related genes *via* induction of *METTL3*, *YTHFD2* and genes involved in transcription elongation	↑ Treg infiltration, ↓ interferon responses, ↓ ICI efficacy	CD133+ patient-derived GSCs	(77)
	CLOCK-BMAL1 complex	Induction of LGMN *via* OLFML3	↑ Infiltration and polarization of immunosuppressive microglia	Patient-derived GSCs	(89, 92)
Chromatin modifications	ATRX deficiency	BRD3/4-dependent induction of PD-L1/2 and immune-suppressive cytokines/chemokines	↑ T cell apoptosis, ↓ T cell-mediated tumor cell lysis, ↑ Macrophage polarization, and ↑ Treg infiltration	IDH-mutant glioma cells	(95)
	HDACs	MHC-1 downregulation	↓ T cell recognition of GSCs, ↓ T cell-mediated GSC lysis	CD133+ patient-derived GSCs	(44)
	MBD3	Promotes assembly of the HDAC-containing NuRD complex which represses STAT1 expression *via* histone deacetylation	↓ interferon-mediated GSC suppression	CD133+ patient-derived GSCs	(106)
DNA methylation	Hypermethylation	↓ NKG2D ligand expression	↓ NK cell-mediated GSC killing	IDH-mutant GSCs	(108)
	Hypermethylation	↓ expression of HLA genes	↓ Immune recognition of GSCs	GSCs induced *via* lentiviral expression of reprogramming TFs in GBM-derived cells	(113)
	Hypermethylation	↓ FAS, MHC-1, and ICAM1 expression	↓ T cell recognition of GSCs, ↓ T cell-mediated GSC lysis	Mouse GSCs	(46)
	Hypomethylation	Enhanced immune evasion *via* activated expression of immune-suppressive genes	↑ Macrophage and MDSC infiltration, ↑ TIL exhaustion	Mouse GSCs & patient-derived GSCs	(68)
RNA methylation	ALKBH5	Upregulation of CXCL8/IL8 *via* demethylation of lncRNA NEAT1	↑ TAM infiltration	U87 GBM cells	(119)

DC, dendritic cell; GSC, glioma stem cell; Treg, regulatory T cell; ICI, immune checkpoint inhibitor; HDACs, histone deacetylases; NK, natural killer; IDH, isocitrate dehydrogenase; MDSC, myeloid-derived suppressor cell; TIL, tumor-infiltrating lymphocyte; TAM, tumor-associated macrophage; TFs, transcription factors. ↓, decreased; ↑, increased.

**Table 2:** Summary of cutting-edge technologies for cell-level resolution analysis.

Sequencing Modality	Description	Detection and Deconvolution Platforms
Bulk RNA-seq deconvolution	Estimated extent of immune/stromal compartments & tumor purity	ESTIMATE (149)
	Estimated proportions of infiltrating immune cell types	CIBERSORTx (150), xCell (151), MCP-counter (152), EPIC (153, 154), quanTIseq (155), TIMER (156)
	Gene expression profiles of imputed cell populations	CIBERSORTx (150)
Single-cell omics	mRNA expression	scRNA-seq
	Chromatin accessibility	scATAC-seq (157)
	DNA methylation	scRRBS (158)
	Surface marker-based quantification of cell population frequencies	CyTOF (159, 160)
	T cell receptor (TCR) sequences	Multiplex PCR- or RACE PCR-based TCR sequencing (161)
	Histone modifications	scCUT&Tag (162, 163)
Same-cell Single-cell Multi omics	Cell surface protein epitopes + mRNA expression	CITE-seq (164, 165)
	Chromatin accessibility + mRNA expression	sci-CAR (166)
	DNA methylation + CNVs + mRNA expression	scTRIO-seq (167, 168)
	Chromatin accessibility + DNA methylation + mRNA expression	scNMT-seq (169)
Spatial omics	Sequencing-based RNA expression imaging	STARmap (170), FISSEQ (171)
	*In situ* hybridization RNA expression imaging	RNAScope (172), MERFISH (173, 174), seqFISH/seqFISH+ (175–177)
	Barcode-based Spot-capture RNA expression	Slide-seq (178, 179), 10X Visium, HDST (180)
	Localization of proteins/metabolites/lipids *via* mass spectrometry imaging	ToF-SIMS (181, 182), MALDI-TOF/-FTICR (183)
	Spatially resolved antibody-based epitope detection	Imaging mass cytometry (184)

## 3 Analytical tools to explore GBM heterogeneity

### 3.1 Deconvolution of bulk tumor sequencing

Whole tumor sequencing modalities, such as whole-genome and whole-exome sequencing, RNA-sequencing (RNA-seq), bisulfite sequencing, and assays for chromatin accessibility sequencing (ATAC-seq), have been widely used to classify tumors and explore context-specific cancer biology. However, these sequencing analyses conducted on dissociated bulk tumor specimens output an average value across all cells, placing limitations on the ability to distinguish which cell type or compartment is contributing to said output and, therefore, interfering with exploration of heterogeneous cell populations and phenotypes. Computational tools to deconvolute bulk-sequencing (bulk-seq) data have been developed, allowing one to take full advantage of large, pre-existing datasets of bulk-seq patient tumor specimens. These tools present an opportunity to infer cellular-level data from a vast number of patient tumors when the use of more advanced technologies is unfeasible or cost-prohibitive.

Computational deconvolution of bulk RNA-seq data has garnered interest in the realm of cancer immunology by granting insight into the composition of the immune compartment (e.g., cell type proportions and overall extent of immune infiltration) and transcriptional phenotypes within immune cell types based on associated expression of cell-specific markers (149–156). The degree of immune cell infiltration and tumor purity (i.e., proportion of tumor composed of neoplastic cells), calculated through ESTIMATE (149), have been associated with molecular phenotypes and trends in patient survival, with high immune cell infiltration and low tumor purity corresponding to mesenchymal tumors and shorter survival (193, 194). More in-depth analytic tools, such as CIBERSORTx (150), xCell (151), MCP-Counter (152), EPIC (153, 154), quanTIseq (155) and TIMER (156), go beyond the extent of immune infiltration and tumor purity to deduce absolute and relative cell proportions based on pre-set or user-defined cell signatures. The CIBERSORTx algorithm takes it a step further by allowing users to impute gene expression profiles (GEPs) for the estimated cell populations. However, this additional step is not recommended for low abundance cell types, such as tumor-infiltrating lymphocytes in GBM, due to insufficient statistical power. In populations with sufficient abundance, imputed GEPs can define cell-type specific transcriptional phenotypes in immune cells (e.g., M1/M2 macrophages, functional or exhausted T cells, activated natural killer or dendritic cells, etc.) and permit further exploration into cell-cell interactions based on ligand/receptor expression within respective cell types.

The value of bulk deconvolution algorithms is ultimately dependent upon two factors: (i) use of a validated, context-specific cell signature matrix and (ii) supportive findings through *in vitro* and/or *in vivo* experimentation. Varn et al. applied the CIBERSORTx platform to construct and validate a glioma cell signature matrix encompassing both neoplastic and non-neoplastic cell types derived from single-cell RNA-seq of IDH-wildtype and IDH-mutant patient gliomas (31). These efforts defined the temporal transitions in the composition of neoplastic cell populations and the associated changes in infiltrating immune cell types in newly diagnosed and recurrent tumors (31, 195). Moreover, a second CIBERSORTx cell signature matrix was constructed to infer proportions of GBM histological features within tumors by using gene expression profiles specific to Ivy GAP-defined histological features (e.g., leading-edge, infiltrating tumor, cellular tumor, microvascular proliferation, and pseudopalisading cells around necrosis) (196). After pathologist-based validation of the inferred proportions, Varn and colleagues were able to calculate correlations between cellular states and histological features. Together, utilization of CIBERSORTx software to estimate proportions of distinct cellular states and phenotypes and the extent of hallmark histological features (196, 197) allowed for comprehensive analysis of tumor cell interactions with and in relationship to the TME in a computational manner, setting the stage for downstream investigation of potentially therapeutically actionable cell states and mechanisms.

While tools for deconvolution of bulk sequencing still have their place in cancer research, especially due to their low cost and accessibility and the large volumes of publicly available data from patient tumors, the advent of cutting-edge single-cell sequencing technologies have turned what was once purely computational predictions into actual omics information at cellular-resolution.

### 3.2 Single-cell tools to explore glioma-immune cell interactions

The development of single-cell technologies to explore cellular states at the genomic, epigenomic, transcriptomic, proteomic, and metabolomic levels has led to unprecedented understanding of the inner workings of cancer (198–200). The resolution of single-cell sequencing allows for in-depth evaluation of rare cancer cell populations (e.g., GSC subsets and tumor-infiltrating immune cell subsets) within tumors that are otherwise lost in bulk analyses. The growing repertoire of single-cell technologies allows us to explore not only the constantly adapting cancer cell population but also the non-neoplastic cells in the surrounding TME, providing insight into the fluctuating & bidirectional communication between cell compartments.

In glioma, single-cell RNA-seq (scRNA-seq) analyses have broadened our knowledge of dynamic cellular and molecular phenotypes that represent the heterogeneous nature of gliomas, confer therapy resistance, and drive tumor recurrence in patients (1, 59, 134). Previously, GBMs were classified by their molecular subtype as determined by bulk sequencing of tumors (43). However, classification of tumor subtypes based on bulk transcriptome analyses masks the inherently high degree of intra-tumoral heterogeneity in GBM, demonstrated in early single-cell analyses by the representation of multiple molecular subtypes within individual tumors (1). Since then, scRNA-seq analyses conducted in patient tumors have defined distinct cellular states that vary in respect to their gene expression profiles (59, 60, 201), interaction with the TME (31, 41, 59, 68), and metabolic states (59, 135). Notably, Neftel et al. demonstrated that glioma cells can exist in and transition between four cellular states, oligodendrocyte-progenitor-like (OPC-like), neural progenitor-like (NPC-like), astrocyte-like (AC-like), and mesenchymal-like (MES-like), that differentially express genes involved in cell cycle, development, metabolism, and immune response, and in which all but one state (MES-like) resemble normal neurodevelopmental cell types (59). Cells in the MES-like state, which expresses higher levels of hypoxia-response and glycolytic genes relative to the other states, form synergistic relationships with TAMs to promote their respective mesenchymal-like, immunosuppressive phenotypes, demonstrated by Hara et al. (41).

Aside from scRNA-seq, one of the more commonly used single-cell techniques in the study of gliomas is cytometry by time-of-flight (CyTOF) (159, 160). CyTOF provides valuable information on cell proportions based on surface marker expression and has been used to characterize variation in immune cell landscapes between primary and metastatic CNS cancers (19), newly diagnosed and recurrent GBMs (20), and immune checkpoint inhibitor (ICI) refractory and responsive cancers and GBM mouse models (202). For example, Fu et al. identified a significant decrease in the proportion of tumor-promoting macrophages and microglia with recurrence whereas the frequencies of T cells, B cells, and NK cells were relatively unchanged (20). In another study, Simonds and colleagues compared immune cell proportions between ICI-refractory (GBM and sarcoma) and ICI-sensitive (renal cell carcinoma) cancers and found decreased T cells and DCs, but increased TAMs in ICI-refractory tumors (202). This aligns with the previously discussed finding that brain metastases, which are more sensitive to immunotherapy than GBM, have relatively more leukocytes and less myeloid cells in their TME, also assessed through CyTOF analysis (19).

Additional insight into cancer-immune interactions from the perspective of infiltrating immune cells can be obtained through techniques like CITE-seq (164, 165) and T cell receptor (TCR) sequencing (161) that provide information on surface marker-defined cell expression profiles and TCR variability and clonality, respectively. The use of CITE-seq, which simultaneously quantifies epitope-specific cell types and their associated transcriptomes *via* RNA sequencing, in glioma has identified novel markers for various transcriptionally defined subsets of TAMs with demonstrated susceptibility to macrophage-directed therapeutics (203). This exploration of the TAM population also unveiled a shift from microglia-derived to monocyte-derived TAMs in the TME with tumor recurrence (203). Notably, high infiltration of monocytic macrophages relative to resident microglia corresponds to increased malignancy in gliomas (19, 204). As for the tumor-infiltrating lymphoid population, TCR sequencing of newly diagnosed and recurrent GBMs revealed a diminished TCR repertoire with recurrence (22), which leads to suppressed T cell-mediated immune response *via* altered antigen-recognition machinery. Based on our understanding of how dynamic GSC states differentially express MHC molecules (44, 46, 113) and the fundamental knowledge that TCR repertoires are shaped in response to antigen exposure, one could infer that immunoediting mechanisms within GSCs directly alter T cell clonality during tumor progression, adding another facet to the immune evasive nature of glioma cells. However, this has yet to be conclusively demonstrated in the context of gliomas. Alternatively, a restricted TCR repertoire could signify T cell specification against the most actionable tumor antigens; however, those T cells are rendered ineffective for the reasons discussed in this review. Hypotheticals aside, these single-cell analyses expand on our understanding of immune TME changes with recurrence that suppress the immune response and facilitate glioma progression.

As discussed previously, epigenetic changes contribute significantly to the acquisition of an immune evasive phenotype in glioma cells in response to immune exposure, a mechanism widely referred to as immunoediting (68). These single-cell modalities to measure epigenetic changes can be combined with single-cell transcriptomics to provide unparalleled information on adaptive cell transitions within tumors. For example, a multimodal approach involving scRNA-seq and single-cell reduced representation bisulfite sequencing (scRRBS) in gliomas by Johnson et al. revealed DNA methylation-mediated induction of cell states associated with activation of stress response pathways and increased therapy resistance (60). Defined by distinct transcriptome and DNA methylome profiles, Johnson and colleagues identified three cell states referred to as stem-like, proliferating stem-like, and differentiated-like. Multi omic analysis revealed key transcriptional regulators in each cell state with differentiated-like cells having enhanced activity in transcription factors involved in hypoxia and stress response, demonstrating the value of epigenetic modifiers in stress-induced cell state plasticity (60).

Another technology amenable to single-cell implementation is the Assay for Transposase-Accessible Chromatin *via* Sequencing (ATAC-Seq (157)). Using this approach Guilhamon and colleagues found three GSC states, reactive, constructive, and invasive, distinguished by their chromatin accessibility profiles. Cells in the reactive state had increased accessibility to the promoters of immune-related genes, include genes associated with Tregs. Alternatively, the invasive GSC state had a demonstrated negative correlation with survival in orthotopic xenografts (201).

Currently, several technologies are being developed for multimodal single-cell application (**Table 2**) in addition to sophisticated computational tools for downstream analyses following single-cell sequencing. Often used in developmental biology but increasingly applied to cancer studies, these analyses can inform cellular hierarchies through pseudo-lineage tracing (e.g., pseudotime analysis (30, 205–207)), predicted cell state transitions (e.g., RNA Velocity (142)), and even metabolic phenotypes (e.g., Compass (208)). Since single-cell sequencing provides a point-in-time view of cell states, these algorithm-based insights into cellular transitions may reveal previously undetectable adaptive mechanisms in GSCs. Multi-plexing these state-of-the-art tools will permit interrogation of highly dimensional cell phenotypes at an unparalleled depth and can provide insight into clonal architecture and lineage tracing, regulators of GSC states, links between genetic and epigenetic profiles, drivers of adaptive responses to therapeutic or immune stimuli, and more (199). We envision these approaches being combined with scRNA-Seq to explore the role of the TME in promoting reversible, epigenetic-mediated GSC state transitions and, thus, build upon similar studies utilizing bulk multimodal analysis in patient-derived GSCs (61). Similar analyses at single-cell resolution would be invaluable in examining specific cell subsets and assessing their reciprocal impact on the TME.

Unfortunately, every rose has its thorn, and a major one for these cutting-edge single-cell sequencing technologies is the complete absence of spatial information. When it comes to understanding the influence of endogenous cell interactions, specifically GSC interactions with the surrounding TME, this information is critical, especially for pre-clinical studies relying on accurate representation of patient tumors. However, this information is lost with dissociative single-cell techniques, which is where newly generated spatially resolved omics technologies come into play.

### 3.3 Spatial omics

While cell-cell interactions can be inferred to a degree through computational manipulation of scRNA-seq data (209–212), spatial omics provide next-level understanding of these interactions by accounting for endogenous tissue organization and cell-cell proximity (213). Interrogation of RNA localization in tissue specimens using spatial transcriptomics techniques, broadly classified as either high-plex RNA imaging or spatial barcoding (199, 213), is arguably the most used spatial analysis as of now. High-plex imaging techniques include *in situ* sequencing (e.g., STARmap (170), FISSEQ (171)) and fluorescent *in situ* hybridization (e.g. RNAScope (172), MERFISH (173, 174), and seqFISH (175–177)) that provide single-cell resolution spatial gene expression with the limitation of relying on predetermined target transcripts and quality imaging instrumentation. On the other hand, spatial barcoding or *in situ* capture techniques, including Slide-seq (178, 179), 10X Visium, and high-definition spatial transcriptomics (180), provide unbiased, transcriptome-wide expression output. However, data is collected at multi-cellular spot resolution which requires additional deconvolution analysis to appropriately distinguish individual cell expression.

Aside from gene expression data, spatially resolved proteomic, metabolomic, and lipidomic information can be gathered from tissue specimens using imaging mass spectrometry or cytometry techniques. Unbiased coverage of biochemical features can be achieved through semi-quantitative mass spectrometry imaging (MSI) methods including time-of-flight secondary ion mass spectrometry (ToF-SIMS) (181, 182) and matrix-assisted laser desorption/ionization (MALDI) time-of-flight (MALDI-TOF) or fourier transform ion cyclotron resonance (MALDI-FTICR) mass spectrometry (183). Alternatively, imaging mass cytometry (IMC) can detect and quantify surface protein profiles using isotope-coupled antibody panels (184) in a method analogous to a spatially resolved CyTOF analysis. While many of the above spatial technologies have yet to be applied in the context of GBM, RNAScope and MSI methods have been utilized with the former identifying expression-based prognostic markers from GBM tissues (214, 215). On the other hand, MSI methods have investigated GBM heterogeneity based on metabolic and proteomic profiles and drug distribution in tumors (131, 216–220).

Integration of spatial and single-cell omics data grants unprecedented understanding of cellular and molecular phenotypes of cancer cells within their native environment. Ravi et. Al. define spatially distinct cellular phenotypes distinguished by transcriptional and metabolic programs through integration of spatial transcriptomics, MALDI-FTICR mass spectrometry, and mass cytometry proteomics (132). These five phenotypes were classified as radial glia, spatial OPC, neural development, reactive hypoxia, and reactive immune. Furthermore, cell phenotypes overlapped with previously established GSC states and were further distinguished by their spatial localization and proximity to immune cell infiltrates. Specifically, the reactive immune cell state, which has similarities to both the MES1 and AC-like states defined by Neftel et al. (59), had significant interaction with both myeloid and lymphoid infiltrating immune cells. Moreover, T cells near reactive-immune cells expressed higher levels of PD-1, representative of T cell exhaustion (132). Overall, multi-modal *in situ* sequencing of sequential tumor tissue slices paints a spatially resolved, complete picture of native cellular states, paving the way for mechanistic validation and identification of potentially translatable therapeutic vulnerabilities within specified cell types.

While single-cell and spatial analyses cannot classify GSCs by their defining functional traits (i.e., self-renewal capacity, multipotency, and tumor propagation capacity), these technologies are advantageous in their own rights for characterization of GSCs. Notably, chromatin accessibility profiles and validated GSC-specific transcriptomes can be used to delineate between GSCs and differentiated GBM cells *in silico* (134, 157, 201, 221–223). Additionally, high resolution omics data provides information at a remarkable depth necessary for proper investigation of dynamic and heterogeneous GSC states. Overall, the degree of insight into dynamic GSC biology afforded by multi-dimensional single-cell and spatial technologies has the potential to elucidate not only the factors that coordinate adaptive and immunosuppressive GSC mechanisms but their associated therapeutic vulnerabilities. Meticulous experimental design and utilization of spatially resolved single-cell technologies could conceivably inform novel treatment approaches capable of eliciting substantial anti-tumor response, a feat yet to be achieved in recurrent GBM.

## 4 Therapeutic opportunities

**Figure 3:**
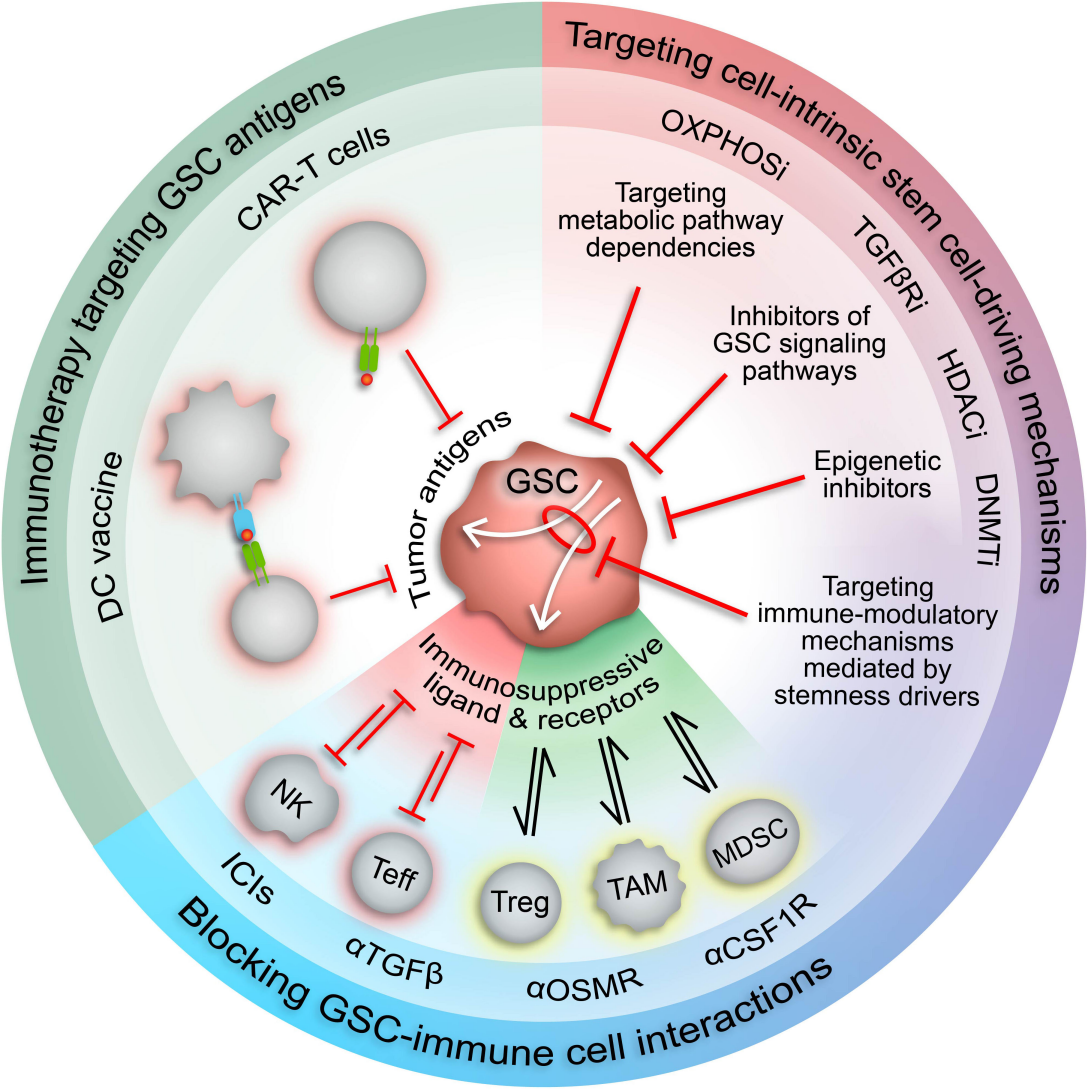
Therapeutic approaches to target immunosuppressive GSCs. Increased understanding of the malignant properties of GSCs and their inherent plasticity and heterogeneity has designated GSCs as desirable therapeutic targets. The role of GSCs in driving and maintaining an immunosuppressive TME suggests that GSC-targeted therapies could potentiate current immunotherapies. Targeting stemness mechanisms that also mediate immunosuppressive mechanisms in GSCs (white arrows) has the potential to augment immunotherapy response in GBM by increasing expression of tumor-specific antigens and repressing immunosuppressive cell interactions. CAR-T cell, chimeric antigen receptor T cell; DC, dendritic cell; NK, natural killer cell; Teff, effector T cell; Treg, regulatory T cell; TAM, tumor-associated macrophage/microglia; MDSC, myeloid-derived suppressor cell; ICIs, immune checkpoint inhibitors; HDACi, HDAC inhibitors; DNMTi, DNMT inhibitors; TGFβRi, TGFβ receptor inhibitors; OXPHOSi, oxidative phosphorylation inhibitors.

The existence of dynamic GSC states shaped by cell-intrinsic factors, the tumor microenvironment, and therapeutic pressures demand a revised approach to GBM therapy development that’s directed at disrupting the bidirectional interactions between GSCs and the TME that promote GBM malignancy, immune evasion, and tumor recurrence. Given the pervasive involvement of epigenetic modifications in GSC plasticity and acquisition of immunosuppressive phenotypes, using epigenetic inhibitors to augment the response of GBM to available immunotherapies warrants investigation (**Figure 3**). Notably, clinical attempts to treat newly diagnosed and recurrent GBM with epigenetic inhibitors (plus chemoradiation and surgery) have had negligible anti-tumor effects compared to standard-of-care treatment alone (186–191). However, preclinical studies investigating the immune effects of epigenetic inhibitors demonstrate increased immunogenicity, indicating that combining epigenetic and immunostimulatory treatment modalities may have potent anti-tumor effects (**Table 3**) (17, 44, 46, 104, 185). Two early phase clinical trials combining anti-PD1 checkpoint inhibitors with azacytidine (DNA methyltransferase inhibitor) or vorinostat (HDAC inhibitor) plus standard chemoradiation are currently underway in IDH-mutant gliomas (NCT03684811) and newly diagnosed GBM (NCT03426891), respectively. Additionally, several clinical trials testing combination epigenetic and immune therapy have been conducted or are currently underway in a variety of cancers, however, durable clinical effects are rare and often restricted to small subsets of patients (224–236). However, ongoing clinical trials may provide insight into mechanisms of resistance and opportunities for therapeutic improvement. Given the knowledge that immune cell states are also intimately controlled by epigenetic modifications (237), the lack of response from combination epigenetic-immune therapies may be due in part to unwanted effects on systemic immune cell pools that alter their phenotypic profiles and potentially induce dysfunctional states. Thus, strategies for targeting immune suppressive mechanism specifically within the TME while avoiding counterproductive systemic effects are needed. Despite these early phase results, the immunogenic effects of epigenetic inhibitors and combo epigenetic-immune therapy in preclinical GBM studies are promising (17, 44, 46, 104, 185, 192) and deserve to be explored to their full extent in clinical trials. Notably, increased immune recognition and resultant cytolysis of tumor cells by T cells and NK cells has been achieved through HDAC inhibitors and DNA hypomethylating agents (e.g., decitabine) (44, 46, 104, 185). Additionally, BRD4 inhibition attenuated an immunosuppressive GSC phenotype to enhance the anti-tumor immune response (17). Furthermore, BRD4 was implicated in an acquired immunosuppressive gene expression profile in GSCs refractory to CAR-T cell treatment. Targeting BRD4 in a mouse model of GBM effectively potentiated CAR-T cell therapy, highlighting the potential for clinical success with combination epigenetic and immunotherapy in GBM (192).

**Table 3:** Epigenetic- and immunotherapy-based attempts to target GSCs.

Epigenetic inhibitors								
Drug	Target	Stage	Trial Phase	Context	Other treatments	Outcome	Ref(s)	NCT #
Decitabine	DNMTs	Pre-clinical	NA	Murine GSCs	NA	Increased T cell-mediated killing of GSCs	(46)	NA
Decitabine	DNMTs	Pre-clinical	NA	Patient-derived primary GBM cell lines	NA	Increased antigen-specific T cell-mediated glioma cytotoxicity	(185)	NA
JQ1	Pan-BET	Pre-clinical	NA	Human GBM neurospheres	NA	Reduced expression of immunosuppressive transcriptome in GSCs; Reduced immunosuppressive effect on T cells & M2 macrophage polarization	(17)	NA
PCI-34051	HDAC8	Pre-clinical	NA	Murine glioma mouse model	NA	Prolonged survival; Reduced invasion of anti-inflammatory microglia; Increased NK cell-mediated glioma cytotoxicity	(104)	NA
Vorinostat	HDACs	Pre-clinical	NA	Murine glioma mouse model	GSC lysate vaccine	Prolonged survival and increased T cell tumor infiltration	(44)	NA
Vorinostat	HDACs	Clinical	I/II	Newly diagnosed GBM	SOC	Did not meet primary efficacy endpoint (OS = 15mo)	(186)	NCT00731731
Vorinostat	HDACs	Clinical	II	Recurrent GBM	Prior SOC	Modest effects; Met primary efficacy endpoint (PFS = 6mo)	(187)	NCT00238303
Vorinostat	HDACs	Clinical	I/II	Recurrent GBM	Bevacizumab, TMZ	Met primary endpoint (PFS = 6mo); Improvement in PFS not statistically significant	(188)	NCT00939991
Vorinostat	HDACs	Clinical	II	Recurrent GBM	Bevacizumab	No significant improvement in PFS or OS	(189)	NCT01266031
Romidepsin	HDACs	Clinical	I/II	Recurrent glioma	Prior SOC	Did not meet primary efficacy endpoint (PFS = 6mo)	(190)	NCT00085540
Panobinostat	HDACs	Clinical	II	Recurrent GBM	Bevacizumab	No significant improvement in PFS	(191)	NCT00859222
**Epigenetic inhibitors plus immunotherapy**								
JQ1 +CAR-T cells	Pan-BET, EGFR	Pre-clinical	NA	Human GBM mouse model	NA	Prolonged survival	(192)	NA
Azacytidine + Nivolumab	DNMTs, PD1	Clinical	I/II	IDH-mutant gliomas	SOC	Completed - No Results	NA	NCT03684811
Vorinostat + Pembrolizumab	HDACs, PD1	Clinical	I	Newly diagnosed GBM	SOC	Active	NA	NCT03426891

SOC, standard-of-care treatment (surgery followed by TMZ & radiation); OS, overall survival; PFS, progression-free survival.

The involvement of GSCs in modulation of the anti-tumor immune response has prompted investigation into GSC-targeted immune-related therapies (**Figure 3**). Proposed methods include chimeric antigen recognition T cells (CAR-T cells) or dendritic cell vaccines directed at GSC-specific surface markers and antigens (53). However, the immunosuppressive effects of GSCs may attenuate the functionality of these engineered immune cells, thereby impeding therapeutic efficacy. Alternatively, forced differentiation of GSCs into a cellular state responsive to current therapeutic regimens holds promise (238), especially in combination with immunotherapy, but fails to consider how controlled differentiation would oppose plasticity mechanisms induced by standard-of-care treatments and/or potentially select for a more immunosuppressive GSC state. Therefore, we propose combining immunotherapy with approaches that disrupt mechanisms governing the transition of GSCs to immunosuppressive states and/or exploit cellular and molecular vulnerabilities inherent in these cell states (**Figure 3**). Identifying actionable therapeutic targets by incorporating high-dimensional, spatially aware single-cell analyses of patient tumor specimens and patient-derived GSC models into preclinical studies allows for development of therapeutics with high potential for clinical success.

## Author contributions

Conceptualization: ALJ, JL, and HL-B.; Writing – Original Draft: ALJ; Writing – Review & Editing: ALJ, JL, and HL-B; Funding Acquisition: JL, and HL-B; Supervision: JL, and HL-B. All authors contributed to the article and approved the submitted version.

## Funding

This study is funded by the National Institute of Neurological Disorders and Stroke (NINDS) grants 1R01NS110087, 1R01NS096754 (JL), 1R01NS120949 (HLB).

## Conflict of interest

The authors declare that the research was conducted in the absence of any commercial or financial relationships that could be construed as a potential conflict of interest.

## Publisher’s note

All claims expressed in this article are solely those of the authors and do not necessarily represent those of their affiliated organizations, or those of the publisher, the editors and the reviewers. Any product that may be evaluated in this article, or claim that may be made by its manufacturer, is not guaranteed or endorsed by the publisher.
